# Purification and characterization of actinomycins from *Streptomyces* strain M7 active against methicillin resistant *Staphylococcus aureus* and vancomycin resistant *Enterococcus*

**DOI:** 10.1186/s12866-019-1405-y

**Published:** 2019-02-19

**Authors:** Manish Sharma, Rajesh Kumari Manhas

**Affiliations:** Department of Microbiology, Guru Nanak Dev University, Amritsar, Punjab 143005 India

**Keywords:** MRSA, VRE, Actinobacterium, Antibacterial, MIC

## Abstract

**Background:**

The increased rate of resistance among two highly concerned pathogens i.e. methicillin-resistant *Staphylococcus aureus* (MRSA) and vancomycin-resistant *Enterococcus* (VRE) necessitates the discovery of novel anti-MRSA and anti-VRE compounds. In microbial drug discovery, *Streptomyces* are well known source of two-thirds of natural antibiotics used clinically. Hence, screening of new strains of streptomycetes is the key step to get novel bioactive compounds with antimicrobial activity against drug resistant bacteria.

**Results:**

In the present study, *Streptomyces antibioticus* strain M7, possessing potent antibacterial activity against different pathogenic bacteria, was isolated from rhizospheric soil of *Stevia rebudiana*. 16S rRNA sequence of M7 (1418 bp) showed 96.47–100% similarity with different *Streptomyces* spp. and the maximum similarity (100%) was observed with *Streptomyces antibioticus* NBRC 12838^T^ (AB184184). Phylogenetic analysis using neighbor joining method further validated its similarity with *Streptomyces antibioticus* NBRC 12838 T (AB184184) as it formed clade with the latter and showed high boot strap value (99%). Antibacterial metabolites isolated from the fermentation broth were characterized using NMR, FT-IR and LC-MS as actinomycins V, X_2_ and D. The purified actinomycins exhibited potent antibacterial activities against test bacteria *viz.* *B. subtilis, K. pneumoniae* sub sp. *pneumoniae*, *S. aureus, S. epidermidis*, *S. typhi, E. coli*, MRSA and VRE. Among these actinomycins, actinomycin X_2_ was more effective as compared to actinomycins D and V. The minimum inhibitory concentration values of purified compounds against a set of test bacterial organisms *viz.* VRE, MRSA, *E. coli* (S1-LF), *K. pneumoniae* sub sp. *pneumoniae* and *B. subtilis* ranged between 1.95 and 31.25 μg/ml.

**Conclusions:**

This study demonstrates that actinomycins V, X_2_ and D produced by *S. antibioticus* strain M7 hold the potential to be used against multidrug resistant bacteria, particularly VRE and MRSA.

**Electronic supplementary material:**

The online version of this article (10.1186/s12866-019-1405-y) contains supplementary material, which is available to authorized users.

## Background

Antimicrobial resistance among microbial pathogens is a significant public health issue, as infections caused by multidrug resistant bacteria take the lives of many people in every year all over the world [1]. Among Gram-positive pathogens, a global pandemic of resistant *Staphylococcus aureus* and *Enterococcus* species currently pose the biggest threat. A single pathogen i.e. methicillin-resistant *Staphylococcus aureus* (MRSA), which was first discovered in 1961, has become a major source of nosocomial and community associated MRSA infections [2, 3]. Clinical isolates of MRSA have high rate of morbidity and mortality as compared to the methicillin susceptible *Staphylococcus aureus* [4, 5]. Also, *Enterococcus faecium* associated with human infections has been developed as multidrug resistant pathogen to vancomycin, ampicillin, and high-levels of aminoglycosides [6, 7].

Vancomycin was the most potent antibacterial drug used against infections caused by MRSA and *Enterococcus*. However, the first case of MRSA exhibiting resistance to vancomycin was reported from Japanese patient in 1996 [8]. According to CDC (Centers for Disease Control and Prevention) April 2013 report, 30% of hospital-acquired infections responsible for 1300 deaths per year were due to vancomycin-resistant *Enterococcus* (VRE) pathogens [9]. While powerful antimicrobial drugs such as synercid, linezolid and daptomycin (lipopeptide) are being used to combat the MRSA and VRE, but some reports showed that these pathogens also have emerged resistance to these effective drugs [10–13]. Because each new antibiotic eventually develops resistance within few years after it is promoted there is always a necessity to find new antimicrobial agents to control antibiotic resistant strains of pathogenic microorganisms.

Recent advances in medical science have sparked to discover the potent therapeutic drugs from the microbial sources. Among microbes, actinobacteria, especially *Streptomyces* spp. are of immense importance as they are known prolific producers of many novel compounds with diverse biological activities [14–17]. Although, nearly two third of the naturally occurring marketed antibiotics are obtained from *Streptomyces* spp. but it is just the tip of the iceberg that have been explored [18]. Therefore, to combat with drug resistance and to discover new therapeutic compounds, we need to screen novel streptomycetes from unexplored resources. Keeping this in mind, we isolated an actinobacterium from rhizospheric soil, exhibiting potent antibacterial activity against multidrug resistant bacteria. The present study reports identification of potent actinobacterium as well as purification and characterization of antibacterial compounds, active against MRSA and VRE, produced by it.

## Methods

### Sample collection

The soil sample was collected into a sterile glass screw cap bottle from the rhizosphere of *Stevia rebudiana* grown in the fields of Palampur, Himachal Pradesh, India**.**

### Test organisms

Different test bacteria such as *Bacillus subtilis* (MTCC 619)*, Escherichia coli* (MTCC 1885), *Klebsiella pneumoniae* sub sp. *pneumoniae* (MTCC 109), *Staphylococcus epidermidis* (MTCC 435), *Salmonella typhi* (MTCC 733), *Mycobacterium smegmatis* (MTCC 6) and *Staphylococcus aureus* (MTCC 96) were procured from Microbial Type Culture Collection (MTCC) and Gene Bank, CSIR-Institute of Microbial Technology (IMTECH), Chandigarh, India. Clinical isolates used in the current study *viz.* *E. coli* (S1-LF) (resistant to cefotaxime, cefoperazone, ciprofloxacin, rifampicin, and clindamycin), MRSA (resistant to methicillin, teicoplanin, imipenem, and clindamycin) and VRE (resistant to vancomycin, methicillin, teicoplanin, imipenem, and clindamycin) were obtained from local hospitals. All the bacterial cultures were maintained on nutrient agar slants in refrigerator at 4 °C.

### Isolation and screening of actinobacteria

Soil sample was air-dried and given the pre-treatment by heating at 100 °C for 1 h to create favorable conditions to accomplish the isolation of actinobacteria. Serial dilution of the treated soil was done up to 10^− 6^. Aliquots of 0.1 ml from 10^− 2^, 10^− 3^, and 10^− 4^ were spread on the surface of SCNA (starch casein nitrate agar) plates. The medium was supplemented with cycloheximide (50 μg/ml) and nalidixic acid (50 μg/ml) to inhibit the growth of fungi and other bacteria, respectively. Plates were then incubated at 28 °C for 7–21 days. Isolated colonies of actinobacteria were sub-cultured and purified on SCNA plates. The isolates were preserved in 20% glycerol at − 20 °C as stock for future use.

### Screening for antibacterial activity

Primary screening was performed by modified method of Kirby Bauer antibiotic susceptibility test using dual culture technique [19]. In this, 6 mm plugs of actinobacteria, grown on SCNA plates, were placed on Mueller Hinton Agar medium (MHA) already seeded with test bacteria. The plates were then incubated at 37 °C. The results as zone of inhibition (mm) were obtained after 24 h of incubation. Isolates which displayed broad spectrum antibacterial activity in ^primary^ screening were subjected to secondary screening using Kirby Bauer agar well diffusion assay [19]. Erlenmeyer flasks (250 ml) containing 50 ml of starch casein nitrate broth were inoculated with 7 days old culture and incubated at 28 °C for 7 days at 180 rpm. The MHA plates seeded with test bacteria (OD equivalent to McFarland standard 0.5) were punctured with sterile cork borer to make wells of 6 mm in size. After addition of culture supernatant (50 μl) to each well, the plates were kept in refrigerator for 1 h for diffusion of active metabolites followed by incubation at 37 °C for 24 h. The results were observed in terms of inhibition zones around the wells. Out of 12 active isolates, strain M7 was selected based on its strong and broad spectrum antibacterial activity.

### Characterization of selected isolate M7

#### Morphological, physiological and biochemical characterization

The culture characteristics of strain M7 were determined according to the International *Streptomyces* Project (ISP) based on the mycelium growth and color, as well as the soluble pigment at 28 °C for 7 days [20]. Melanin production was detected by growing on ISP6 and ISP7 media. Morphological characteristics of the strain, grown on SCNA at 28 °C for 4 days, were observed using bright field light and scanning electron microscopy [21]. Physiological and biochemical tests, like growth at different temperatures, pH, salt concentration, and ability to produce different hydrolytic enzymes were performed as per standard protocols [22–24]. Analysis of the sugar components in whole cell hydrolysate and isomer of diaminopimelic acid (DAP) in the cell wall was done according to the method given by Lechevalier and Lechevalier [25]. Assimilation of sugars as carbon sources was studied according to Shirling and Gottlieb [20].

#### 16S rRNA gene amplification and phylogenetic analysis

DNA extraction from isolate M7 was performed using standard protocol described by Marmur [26]. Using genomic DNA as template, 16S rRNA gene was amplified using universal primers f27 (5’AGAGTTTGATCATGGCTCAG 3′) and r1492 (5’ TACGGCTACCTTGTTACGACTT-3′) [27]. The 1.5 kb PCR product was then got sequenced from IMTECH, Chandigarh (India). The pairwise sequence alignment of 16S rRNA gene sequence was done using ClustalW program and compared with the other related *Streptomyces* spp. retrieved from EzTaxon server (http:// eztaxon-e.ezbiocloud.net) [15]. Neighbor joining method was used to construct phylogenetic tree based on bootstrap values (1000 replications with MEGA6 software) [28, 29]. The 16S rRNA gene sequence (1418 bp) was deposited in GenBank with accession no. KY548390.

### Antibacterial activity profile of *Streptomyces* strain M7

Production of active metabolites was done by carrying out fermentation in Erlenmeyer flasks (250 ml), containing 50 ml of production medium (SCN broth) inoculated with 2% inoculum, at 28 °C for 10 days under agitation at 180 rpm. After every 24 h, the flasks were harvested and the biomass was separated from the culture broth by centrifugation at 10,000 rpm for 20 min. The biomass was dried at 60 °C for 2 days, weighed and expressed in mg on dry weight basis. The remaining cell free culture supernatant was used to check the antibacterial activity against test bacterial cultures using agar well diffusion assay.

### Extraction of active compounds

For the recovery of antibacterial metabolites, 96 h old culture supernatant was extracted twice with different organic solvents *viz.* ethyl acetate, chloroform, hexane, butanol and diethyl ether using solvent-solvent extraction technique. The separated organic phase was concentrated using the rotary evaporator and redissolved in respective solvent and checked for its antibacterial activity against *B. subtilis.*

### Bioautography

For the analysis of antibacterial metabolites, the ethyl acetate extract was separated by thin layer chromatography (TLC) using ethyl acetate: hexane (9:1, *v*/v) as solvent system and the developed chromatogram was observed under UV light and in iodine chamber. TLC strips were then aseptically placed on the surface of MHA already seeded with the test bacterium. Then, the plates were kept at 4 °C for 1 h to allow diffusion of the active metabolites from the TLC strips. After that the plates were incubated at 37 °C for 24 h and observed for the presence of inhibition zones which indicate the number of active compounds in the solvent extract.

### Purification of the active compounds

To purify the antibacterial compounds, ethyl acetate extract (150 mg) was subjected to silica gel chromatography. The column (35 × 1.0 cm) was packed with silica gel (60–120 mesh) using hexane as solvent and eluted step-wise with 100% hexane, 90:10, 80:20, 70:30, 60:40, 50:50, 40:60, 30:70, 20:80, 10:90 (*v*/v) of hexane: ethyl acetate, 100% ethyl acetate (200 ml each) at a flow rate of 2 ml/min. A total of 88 fractions of 25 ml each were collected, concentrated and redissolved in the same solvent ratio from which they were recovered. Fractions showing antibacterial activity against *B. subtilis* were pooled and further purified using size exclusion chromatography with Toyopearl resin HW-40 and methanol as an eluent. A total of 65 fractions were collected and screened for antibacterial activity against *B. subtilis.* Active fractions were further fractionated using preparative RP-HPLC: Shimadzu MicrosorbMV, 100 mm × 10 mm ID, 10 μm, at a flow rate of 3 ml/min, with mobile phase of acetonitrile: H_2_O (55%) in 30 min and UV detection at 440 nm. The peaks of the chromatogram were collected by using a fraction collector attached with the HPLC system, concentrated and then screened for antibacterial activity.

### Structure elucidation of the purified compounds

The structures of the bioactive compounds were elucidated using various spectroscopic techniques. Physicochemical properties such as appearance, color, odor and solubility were determined according to the standard procedures [30]. The UV-Visible spectrum was recorded qualitatively on UV- Visible Spectrophotometer (Shimadzu) in the range of 200–800 nm using chloroform as reference solvent. The mass spectrometry (MS) was done with Bruker MICROTOF II spectrometer, Fourier transformation infrared spectroscopy (FT-IR) was recorded with Perkin–Elmer Spectrum RX-IFTIR spectrophotometer in the range 400–4000 cm^− 1^ and nuclear magnetic resonance (NMR) spectroscopy was recorded in chloroform-d [99.8 atom% D, containing 0.1% (*v*/v) tetramethylsilane (TMS)] at 25 °C on 500 MHz AVANCE III Bruker spectrometer equipped with a 5 mm double channel solution state probe [31, 32].

### Antibacterial activity of purified compounds

The antibacterial activity of the purified compounds was assayed using the standard Kirby-Bauer disc diffusion method. Petri plates containing MHA were swabbed with test bacteria and then discs loaded with 25 μg of purified compounds were placed on the surface of the medium followed by compound diffusion at refrigeration temperature for 30 min. The plates were incubated overnight at 37 °C and the zones of inhibition were measured in millimetres.

### Bioautography of purified compounds

The pure compounds (30 μg) were loaded onto pre-coated TLC plates and separated using ethyl acetate-hexane (9:1, *v*/v). The dried TLC plates were placed onto the medium seeded with *B. subtilis*. These plates were kept in refrigerator for 1 h for diffusion. Thereafter, these plates were incubated overnight at 37 °C for 24 h, observed for clear zones and Retardation factor (Rf) values of purified compounds calculated.

### Minimum inhibitory concentration (MIC) of purified compounds

Minimum inhibitor concentrations of the purified compounds were determined by 96 well microtiter plate dilution assay. The different concentrations of the purified compound (0.96, 1.97, 3.95, 7.56, 15.12, 31.25, 62.5, 125 μg/ml) were prepared in sterile water [33] and added to the test bacteria *viz.* VRE, MRSA, S1-LF, *K. pneumoniae* sub sp. *pneumoniae* and *B. subtilis* grown to logarithmic phase (between 0.3 to 0.5 OD at 595 nm). Bacterial culture (100 μl) was mixed with 100 μl of different concentrations of compounds, control blanks contained 100 μl of test compound of different concentrations with 100 μl of nutrient broth, positive control well consisted of 100 μl of bacterial culture and 100 μl of Nutrient broth (NB), negative control contained 200 μl of NB only and the plates were incubated at 37 °C and OD was measured at 595 nm at 24 and 48 h using ELISA microplate reader (Bio-rad, Model 680XR). MIC values were calculated by comparing the growth in wells containing extract to the growth in control wells and are the lowest concentration that resulted in 90% inhibition in growth compared to the growth in control well.

## Results

### Isolation and screening

Out of 20 different actinobacteria isolates recovered from the soil, 12 isolates showed activity against one or more test bacteria in the primary screening. Among these, 7 isolates displayed antibacterial activity in fermentation broth with varying degree of inhibition against different test bacteria. Isolate M7 exhibiting potent antibacterial activity against all the test bacteria was selected for further studies.

### Identification and characterization of strain M7

**Fig. 1:**
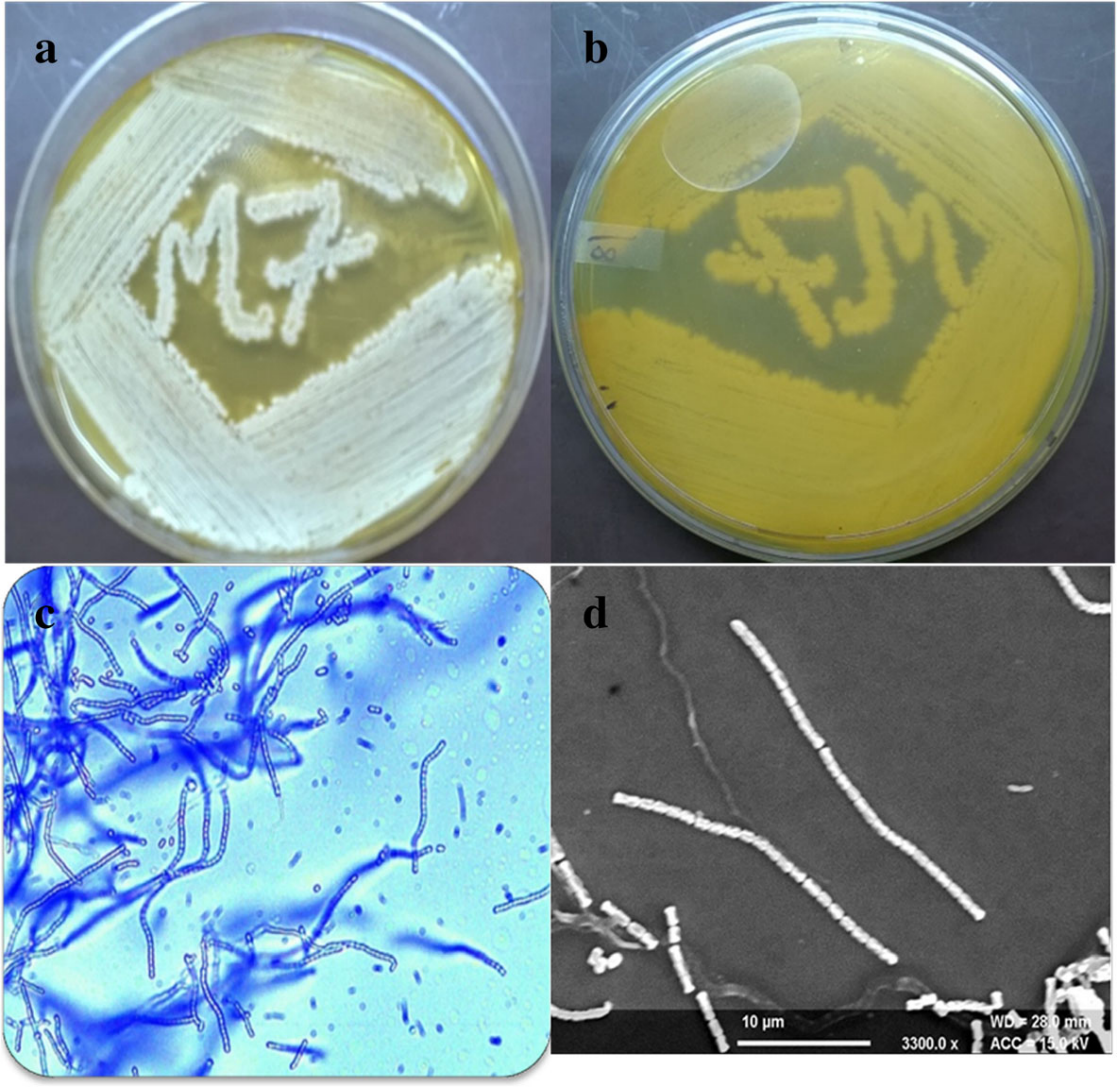
Cultural characteristics of *S. antibioticus* strain M7 **(a)** Aerial Mycelium, (**b)** Substratum Mycelium, (**c)** Aerial hyphae bearing straight spore chains under Light Microscope (100X), (**d)** Scanning electron micrograph (3500X)

The actinobacterial strain M7 grew well on all the ISP media and SCNA with different cultural characteristics (Additional file 1: Table S1). The strain produced yellow colored diffusible pigment on SCNA (Fig. 1a, b) and brown colored pigment on ISP6 and ISP7 media. Microscopic studies showed the formation of branched substratum mycelium and rectiflexibilis-type spore chains, bearing 25–38 smooth cylindrical spores on aerial mycelium (Fig. 1c, d). Chemotaxonomic analysis of cell wall and whole cell hydrolysates revealed the presence of type 1 cell wall, containing LL-DAP as the diagnostic amino acid and no characteristic sugar. The physiological and biochemical characteristics of the strain are shown in Table 1. The strain M7 was able to grow at temperature between 25 and 45 °C (optimum at 28 °C), pH 5 to 10 (optimum at pH 7.0) and could tolerate NaCl upto 5%. M7 strain utilized different tested carbon sources: starch, glycerol, D-glucose, sucrose, lactose, D-fructose, raffinose, inositol, arabinose and D-xylose. The maximum growth was observed on medium containing starch, maltose and sucrose. Biochemical studies showed positive results for production of catalase, oxidase, hydrogen sulfide and citrase, the results for methyl red, voges-proskauer, indole and nitrate reduction were negative. Strain was also able to hydrolyze starch, cellulose, esculin, urea, lipid, and gelatin but did not show casein hydrolysis.

**Table 1:** Morphological, Biochemical and Chemotaxonomic Characteristics of *Streptomyces antibioticus* M7

Characteristics Results	
Cultural Characteristics	
Spore mass	Grey
Spore chain	Straight
Spore shape	Cylindrical
Sugar pattern	No sugar
Substratum mycelium	Yellow
Aerial mycelium	White
Diffusible pigment	Yellow
Diaminopimelic acid	LL-DAP
Physiological characteristics	
Salt tolerance	5.0%
Temperature tolerance	20 °C to 45 °C
pH tolerance	5–10
Production of Melanoid pigment	
Tyrosine agar medium	+
Peptone Yeast extract agar medium	+
Biochemical characteristics	
Indole production	–
Methyl red	–
Vogues proskaur test	–
Citrate utilization	+
Casein hydrolysis	–
Catalase test	+
Urea hydrolysis	+
Esculin hydrolysis	+
Starch hydrolysis	+
Lipid hydrolysis	+
Gelatin hydrolysis	+
Hydrogen sulphide test	+
Oxidase test	+
Nitrate Reduction test	–
Utilization of Sugar	
Maltose	+
D-Glucose	+
Sucrose	+
Lactose	+
Inositol	+
D-Xylose	+
D-Fructose	+
Raffinose	+
Arabinose	+
Starch	+
Glycerol	+

+ = Positive, − = Negative

**Fig. 2:**
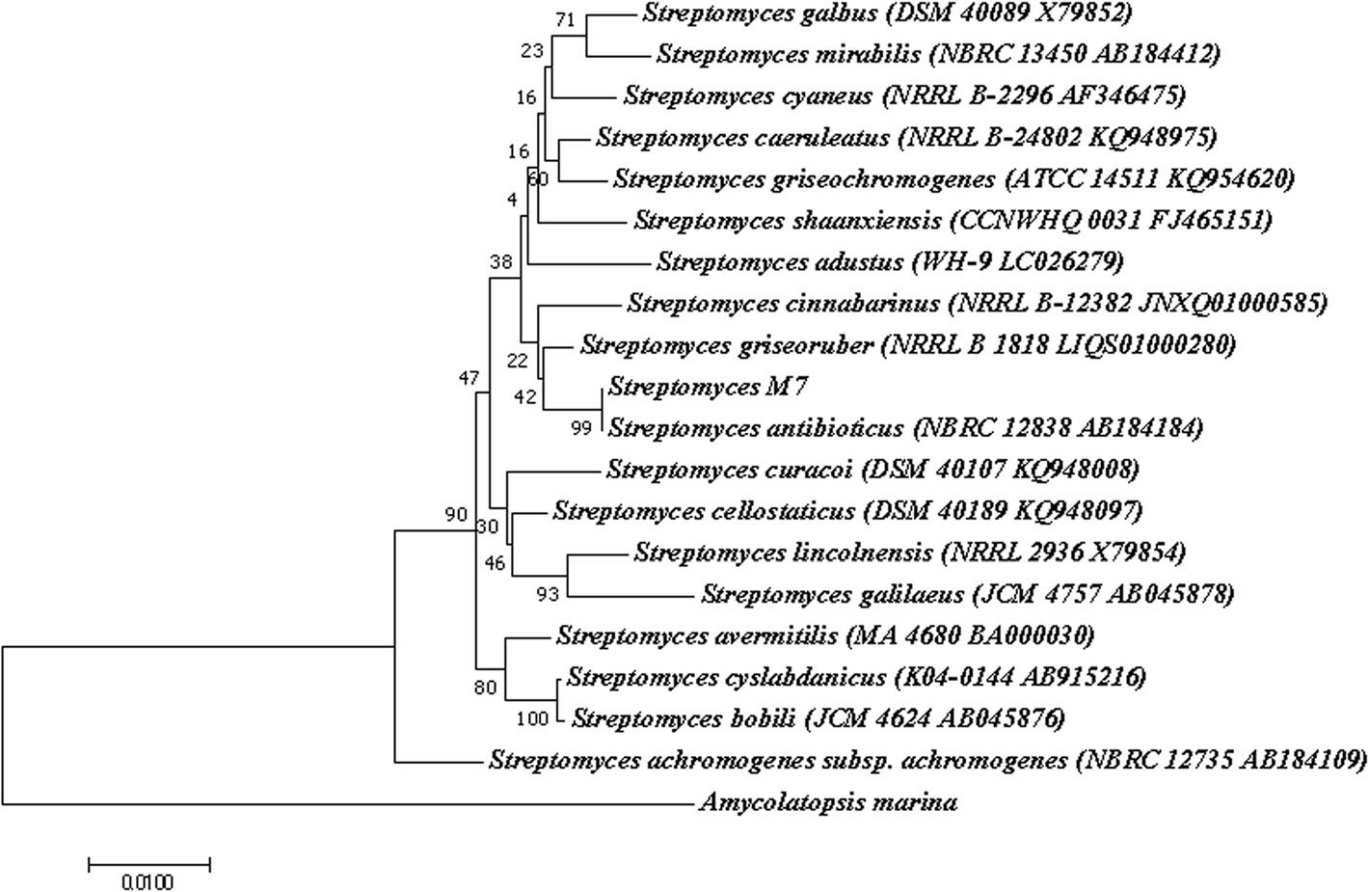
Phylogenetic tree obtained by neighbor joining analysis of 16S rRNA gene sequences showing the relationship between M7 and related species belonging to genus *Streptomyces* obtained from EzTaxon database*.* Numbers on branch nodes are bootstrap values (expressed as percentage of 1000 replications)

Based on morphological, cultural and chemotaxonomy characteristics, isolate M7 was designated as *Streptomyces* sp. and was further confirmed by 16S rRNA sequencing. Alignment of 16S rRNA sequence of M7 (1418 bp), using EzTaxon database [15], showed 96.47–100% similarity with different *Streptomyces* spp. It showed the maximum similarity (100%) with *Streptomyces antibioticus* NBRC 12838^T^ (AB184184) with overlapping of 1417 bp out of 1418 bp. Phylogenetic tree constructed by neighbor joining method further confirmed its similarity with *S. antibioticus* (with high bootstrap values 99%) (Fig. 2).

### Antibacterial activity profile of *Streptomyces* strain M7

**Fig. 3:**
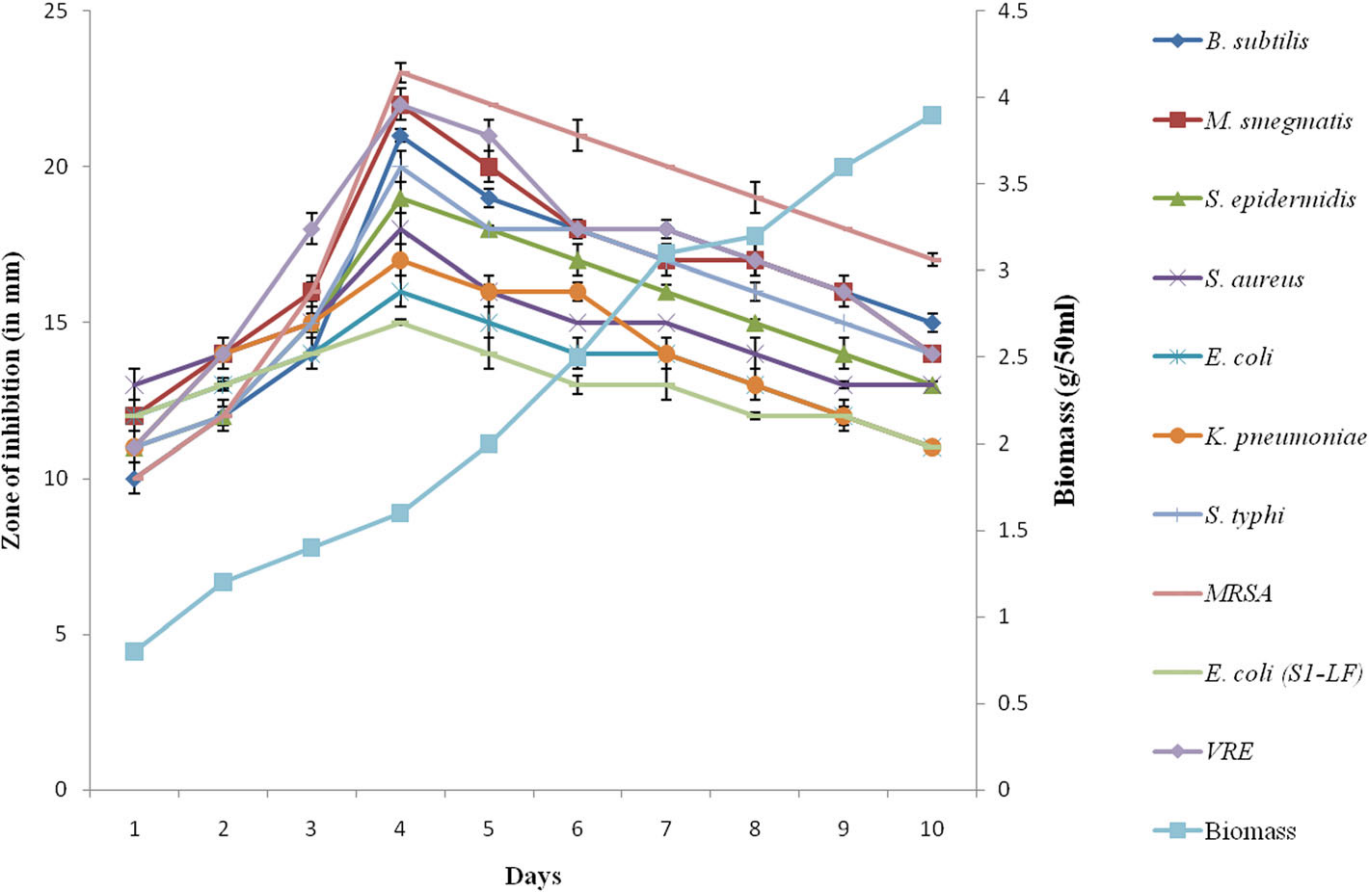
Growth and antibacterial activity of *S. antibioticus* strain M7 against test organisms *viz. B. subtilis, M. smegmatis, K. pneumoniae* sub sp. *pneumoniae*, *S. aureus, S. epidermidis*, *S. typhi, E. coli, E. coli* (S1LF), MRSA and VRE

In vitro bioassay demonstrated strong antibacterial activity of *Streptomyces* strain M7 against tested bacteria. It showed pronounced inhibition against pathogenic bacteria *viz.* VRE, MRSA and *M. smegmatis* with inhibition zones of 23–21 mm*.* Moderate to weak activity was observed against *B. subtilis, K. pneumoniae* sub sp. *pneumoniae*, *S. epidermidis*, *S. typhi, E. coli,* S1-LF and *S. aureus* with inhibition zones of 15–20 mm. This suggests that MRSA, VRE and *M. smegmatis* are more susceptible as compared to other test bacteria. The production of active metabolites in SCN culture broth started after 24 h of incubation, reached the maximum after 96 h and declined slightly as the incubation was further extended (Fig. 3).

### Recovery, separation and bioautography of bioactive metabolites

Among all the solvents used, ethyl acetate was found to be the best solvent to achieve the maximum recovery of active metabolites from fermentation broth of pH 5.0. The extracted metabolites in ethyl acetate were concentrated under reduced pressure using rotary evaporator and resulted orange colored dried extract was redissolved in ethyl acetate. Separation of antibacterial metabolites present in crude solvent extract was carried out by thin layer chromatography using ethyl acetate: hexane (9:1, *v*/v) as solvent system (Additional file 2: Figure S2a**)**. Bioautography of purified actinomycins also confirmed the three antibacterial compounds with Rf values of 0.25 (compound P1), 0.52 (compound P2), and 0.48 (compound P3) (Additional file 2: Figure S2b**).**

### Purification of antibacterial compounds from *S. antibioticus* strain M7

For purification of antibacterial compounds, fermentation was carried out in SCN broth for 4 days at 28 °C. After 4th day of incubation, culture broth was centrifuged at 10,000 rpm and then extracted twice using ethyl acetate (1:2, *v*/v). The obtained orange color crude extract was subjected to silica gel column chromatography for isolation of active compounds. Twenty six fractions (33–58), eluted with hexane: ethyl acetate (10:90, v/v) showed antibacterial activity*.* These were pooled together based on their similar TLC pattern and concentrated. The pooled fraction was further fractionated on size exclusion chromatography using toyopearl resin HW-40. Nine fractions (27–35) which showed activity were again pooled and finally subjected to semi-preparative HPLC. Individual peaks were collected, and antibacterial activity was detected in two peaks with retention times of 10.524 and 15.443 min (Additional file 2: Figure S1a). Active peak with retention time of 15.443 was further fractionated by performing HPLC using acetonitrile: water (95:5, v/ v) as gradient and resulted in separation of compounds P2 and P3 with retention times of 5.814 and 6.548, respectively (Additional file 2: Figure S1b). The collected peaks were further chromatographed using acetonitrile: water (55:45) and single peaks with retention times of 10.628, 15.318, and 15.999 min were obtained which indicated the purity of the compounds **[**Additional file 2: Figure S1 (c-e)].

### Characterization of the purified antibacterial compounds

**Fig. 4:**
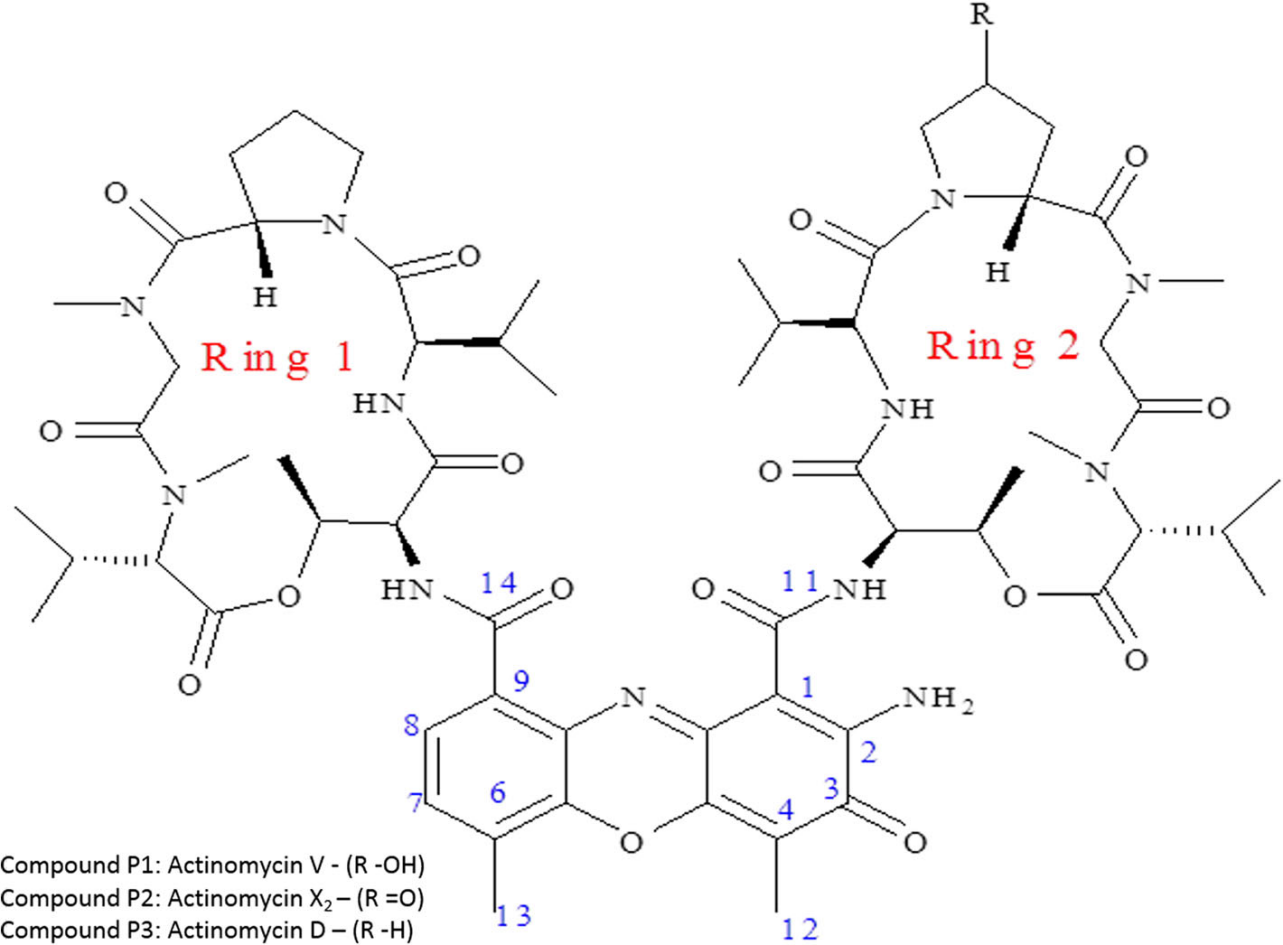
Chemical structure of Actinomycin V (P1), Actinomycin X_2_ (P2), Actinomycin D (P3) from *S. antibioticus* strain M7

**Fig. 5:**
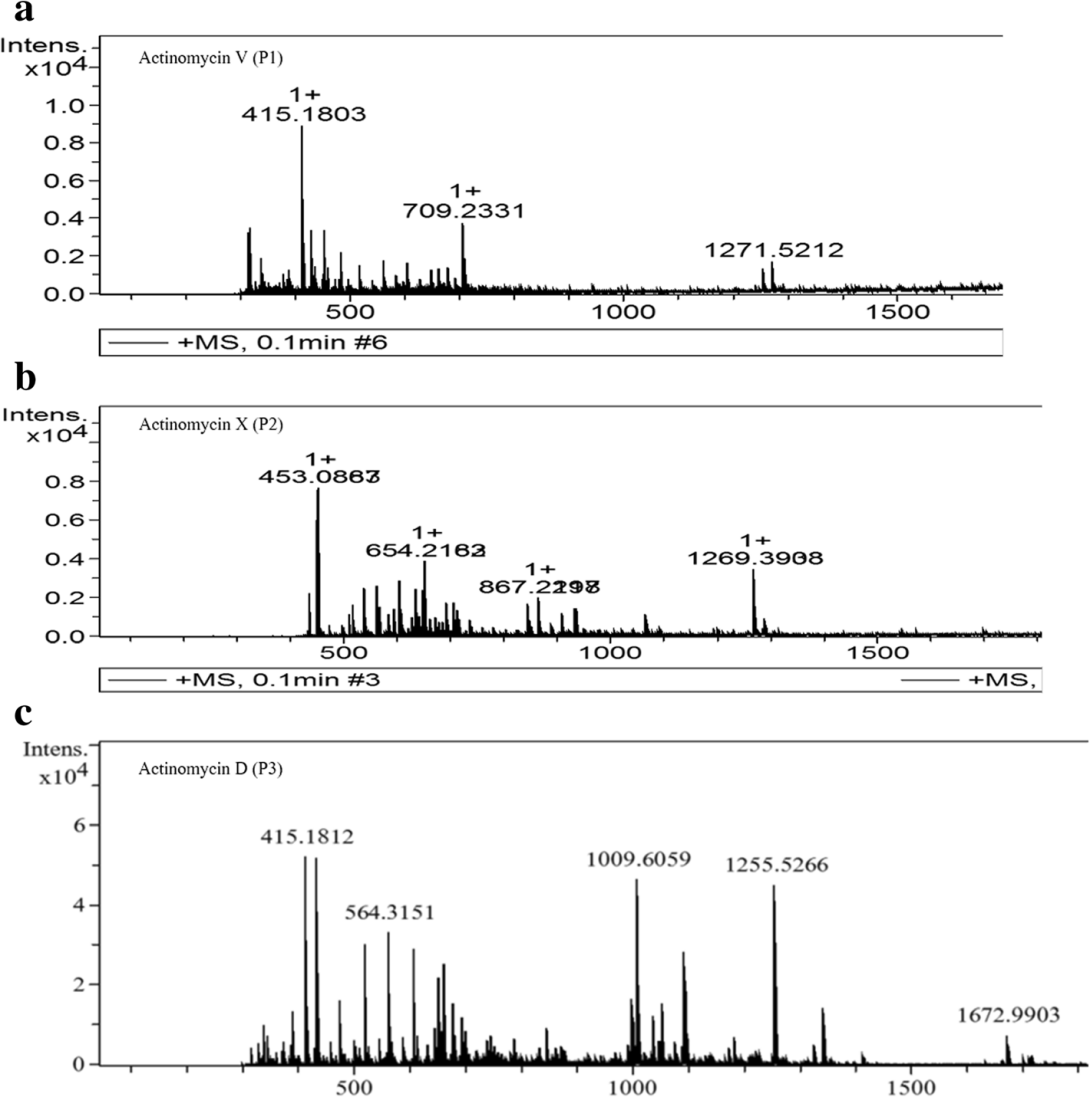
Mass Spectra of purified compounds from *S. antibioticus* strain M7 (**a)** Actinomycin V (P1), (**b)** Actinomycin X_2_ (P2), (**c)** Actinomycin D (P3)

The three active compounds (P1, P2 and P3) were characterized as actinomycins by various spectrometric techniques such as UV-visible, FT-IR, Mass spectrometry and ^1^H NMR (Fig. 4). All the compounds were soluble in water, chloroform, methanol, ethyl acetate and DMSO. The FT-IR data of these purified compounds confirmed the presence of various functional groups such as primary amine, hydroxyls, alkenes, primary amide and carbonyl groups which are the characteristics of phenoxazone ring. The presence of band range of 2854–2874 cm^− 1^ and 2956–2964 cm^− 1^ showed the symmetrical and asymmetrical C-H stretching of –CH_2_ group, respectively. The Compound P1 (yield: 30 mg) was yellow in color, UV λ_max_ 256, 445 nm, Mass Spectrum (TOF, ESI): *m*/*z* (M^+^): 1271.5212 **(**Fig. 5a**)**, FT-IR (Additional file 2: Figure S3a). From these data along with ^1^H-NMR [Table 2 and Additional file 3: Figure S4 (a-d)] the compound P1 was identified as Actinomycin V. The Compound P2 was orange in color, yield: 30 mg having UV λ_max_ 254, 444 nm, Mass Spectrum (TOF, ESI): *m*/*z* (M^+^): 1269.3968 **(**Fig. 5b**)**, FT-IR (Additional file 2: Figure S3b) and ^1^H-NMR spectrum [Table 3 and Additional file 3: Figure S5 (a-f**)]** identified the compound as Actinomycin X_2_. Similarly, the yellow color compound P3 (yield: 40 mg) was identified as Actinomycin D on the basis of UV λ_max_ 256, 442 nm, Mass Spectrum (TOF, ESI): *m*/*z* (M)^+^: 1255.5266 (Fig. 5c), FTIR (Additional file 2: Figure S3c), ^1^H-NMR spectrum [Table 4 and Additional file 3: Figure S6 (a-g)]. The molecular formulae for actinomycins V, X_2_ and D, calculated on the basis of the NMR, LC-MS and FT-IR data, are C_62_H_86_N_12_O_17_, C_62_H_84_N_12_O_17_ and C_62_H_86_N_12_O_16_, respectively which are also in agreement with previous findings.

**Table 2:** ^1^H NMR Data of Purified Actinomycin V

Group	Compound P1: Actinomycin V (δ-value)		Actinomycin V (Wang et al. 2017) (δ-value)	
Phenoxazone	7.64 (d)		7.64 (d)	
	7.37 (d)		7.35 (d)	
	2.56 (s)		2.54 (s)	
	2.25 (s)		2.22 (s)	
Amino acids	Ring 1	Ring 2	Ring 1	Ring 2
Threonine	4.77 (dd)	4.73 (dd)	4.81 (dd)	4.49 (dd)
	5.19 (m)	5.24 (m)	5.24 (m)	5.24 (m)
	1.27 (s)	1.24 (d)	1.28 (d)	1.24 (d)
	7.37 (d)	7.48 (d)	7.44 (d)	7.50 (d)
Valine	3.57 (dd)	3.55 (dd)	3.57 (dd)	3.55 (dd)
	2.17 (m)	2.07 (m)	2.16 (m)	2.12 (m)
	0.96 (d)	0.94 (d)	0.96 (d)	0.94 (d)
	0.74 (d)	0.74 (d)	0.72 (d)	0.74 (d)
	8.17 (d)	8.01 (d)	8.17 (d)	7.91 (d)
Proline	5.95 (d)	6.02 (d)	5.97 (d)	6.05 (d)
	1.84 (m), 2.78 (m)	4.13 (m), 3.95 (m)	1.84 (m), 2.78 (m)	4.13 (m), 3.95 (m)
	2.06 (m), 2.26 (m)	4.70 (m)	2.06 (m), 2.26 (m)	4.70 (m)
	3.83 (m), 3.77 (m)	3.56 (m), 3.08 (m)	3.85 (m), 3.73 (m)	3.56 (m), 3.08 (m)
Sarcosine	4.62 (d)	4.50 (d)	4.72 (d)	4.55 (d)
	3.63 (d)	3.59 (d)	3.63 (d)	3.59 (d)
MethylValine	2.88 (s)	2.88 (s)	2.87 (s)	2.87 (s)
	2.70 (d)	2.68 (d)	2.71 (d)	2.67 (d)
	2.78 (m)	2.65 (m)	2.78 (m)	2.64 (m)
	0.96 (d)	0.94 (d)	0.96 (d)	0.94 (d)
	0.74 (d)	0.74 (d)	0.75 (d)	0.74 (d)
	2.94 (s)	2.91 (s)	2.91 (s)	2.94 (s)

**Table 3:** ^1^H NMR Data of Purified Actinomycin X_2_

Group	Compound P1: Actinomycin X_2_ (δ-value)		Actinomycin X_2_ (Wang et al. 2017) (δ-value)	
Phenoxazone	7.61 (d)		7.60 (d)	
	7.37 (d)		7.35 (d)	
	2.56 (s)		2.55 (s)	
	2.25 (s)		2.24 (s)	
Amino acids	Ring 1	Ring 2	Ring 1	Ring 2
Threonine	4.55 (m)	4.48 (m)	4.55 (m)	4.48 (m)
	5.15 (m)	5.24 (m)	5.15 (m)	5.24 (m)
	1.14 (d)	1.26 (d)	1.14 (d)	1.26 (d)
	7.17 (d)	7.67 (d)	7.17 (d)	7.67 (d)
Valine	3.57 (dd)	3.71 (m)	3.57 (dd)	3.70 (m)
	2.10 (m)	2.09 (m)	2.10 (m)	2.09 (m)
	0.90 (d)	0.89 (d)	0.90 (d)	0.89 (d)
	1.12 (d)	1.15 (d)	1.12 (d)	1.15 (d)
	7.68 (d)	8.21 (d)	7.68 (d)	8.19 (d)
Proline	5.96 (d)	6.02 (d)	5.95 (d)	6.05 (d)
	1.82 (m), 2.75 (m)	3.85 (m), 2.23 (m)	1.84 (m), 2.75 (m)	3.85 (m), 2.33 (m)
	2.25 (m)		2.24 (m)	
	3.89 (m), 3.74 (m)	4.55 (m), 3.89 (m)	3.85 (m), 3.73 (m)	4.55 (m), 3.89 (m)
Sarcosine	4.72 (d)	4.57 (d)	4.72 (d)	4.57 (d)
	3.62 (d)	3.62 (d)	3.62 (d)	3.62 (d)
MethylValine	2.89 (s)	2.90 (s)	2.88 (s)	2.89 (s)
	2.68 (m)	2.68 (m)	2.68 (m)	2.68 (m)
	2.68 (m)	2.68 (m)	2.68 (m)	2.68 (m)
	0.94 (d)	0.97 (d)	0.94 (d)	0.97 (d)
	0.74 (d)	0.73 (d)	0.74 (d)	0.73 (d)
	2.92 (s)	2.93 (s)	2.92 (s)	2.93 (s)

**Table 4:** ^1^H NMR Data of Purified Actinomycin D

Group	Compound P3: Actinomycin D (δ-value)		Actinomycin D (Wang et al.2017) (δ-value)	
Phenoxazone	7.60 (d)		7.64 (d)	
	7.34 (d)		7.37 (d)	
	2.53 (s)		2.56 (s)	
	2.22 (s)		2.25 (s)	
Amino acids	Ring 1	Ring 2	Ring 1	Ring 2
Threonine	4.59 (d)	4.48 (d)	4.60 (d)	4.48 (d)
	5.21 (d)	5.15 (d)	5.20 (d)	5.16 (d)
	1.23 (s)	1.23 (s)	1.26 (s)	1.26 (s)
	7.13 (d)	7.72 (d)	7.19 (d)	7.81 (d)
Valine	3.52 (m)	3.54 (m)	3.54 (m)	3.55 (m)
	2.18 (m)	2.02 (m)	2.16 (m)	2.08 (m)
	0.97 (d)	0.87 (d)	0.90 (d)	0.89 (d)
	1.10 (d)	1.10 (d)	1.12 (d)	1.12 (d)
	8.14 (d)	7.98 (d)	8.09 (d)	7.94 (d)
Proline	5.99 (d)	5.93 (d)	6.02 (d)	5.98 (d)
	1.88 (m), 2.67 (m)	1.87 (m), 2.67 (m)	1.88 (m), 2.67 (m)	1.87 (m), 2.67 (m)
	2.18 (m)	2.15 (m),	2.17 (m), 2.25 (m)	2.15 (m), 2.25 (m)
	3.73 (m)	3.93 (m)	3.72 (m)	3.82 (m)
Sarcosine	4.75 (d)	4.68 (d)	4.76 (d)	4.70 (d)
	3.62 (d)	3.64 (d)	3.61 (d)	3.64 (d)
	2.88 (s)	2.88 (s)	2.88 (s)	2.88 (s)
MethylValine	2.68 (m)	2.68 (m)	2.67 (m)	2.67 (m)
	0.93 (d)	0.86 (d)	0.96 (d)	0.95 (d)
	0.72 (d)	0.72 (d)	0.75 (d)	0.75 (d)
	2.85 (s)	2.91 (s)	2.90 (s)	2.94 (s)

### Antibacterial activity of purified compounds

**Fig. 6:**
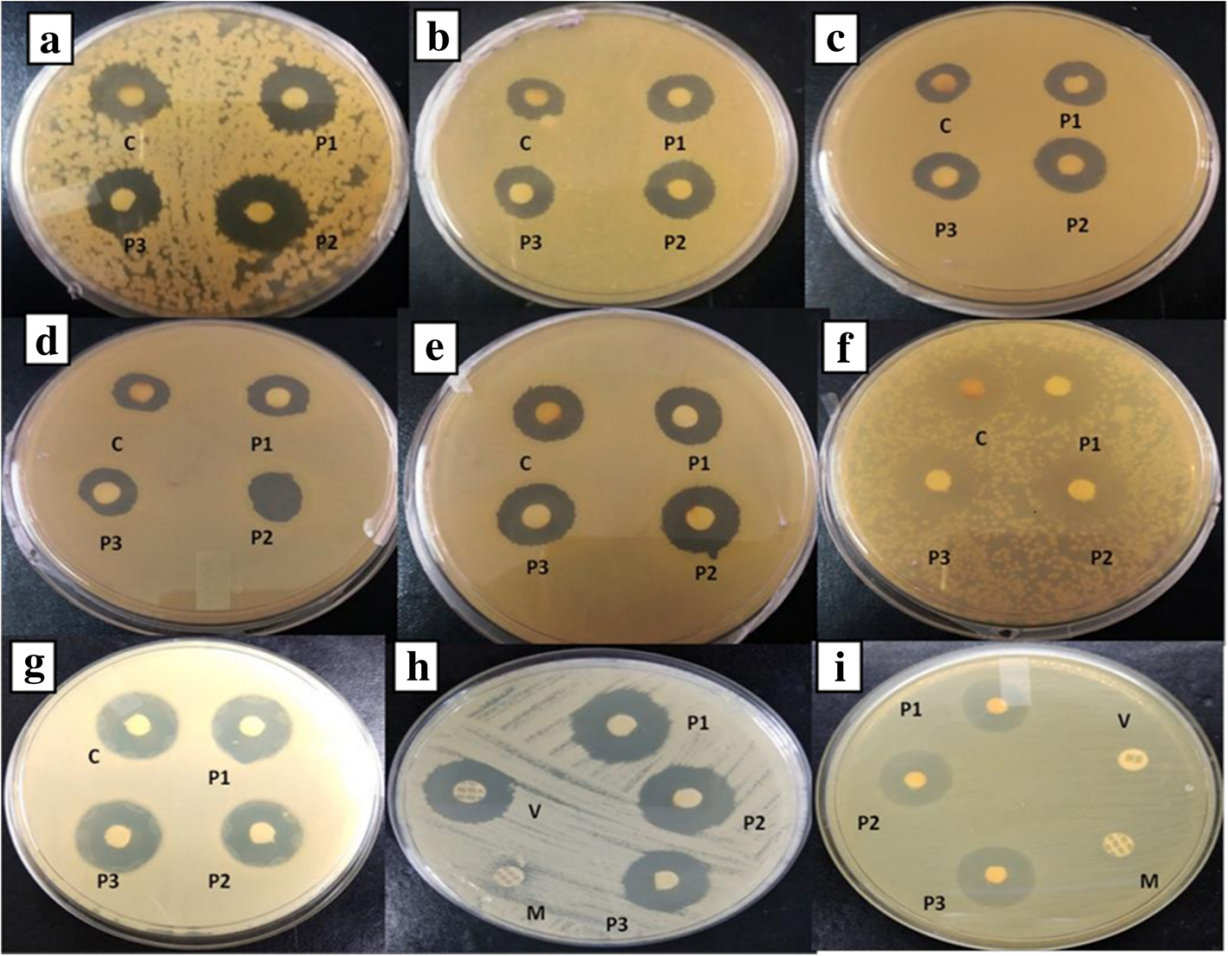
Antibacterial activity of *Streptomyces* strain M7 against pathogenic bacteria: (**a)** *Staphylococcus epidermidis* (**b)** *Salmonella typhi* (**c)** *Bacillus subtilis* (**d)** *Klebsiella pneumoniae* sub sp. *pneumoniae* (**e)** *Staphylococcus aureus* (**f)** *E. coli* (**g)** S1LF (**h)** MRSA (**i)**VRE.C: crude extract, P1: Actinomycin V, P2: Actinomycin X_2_, P3: Actinomycin D, V: Vancomycin (30 μg/disc), M: Methicillin (10 μg/disc)

The purified compounds exhibited potent antibacterial activity against a range of both Gram negative and Gram positive bacteria *viz.* *B. subtilis, K. pneumoniae* sub sp. *pneumoniae*, *S. aureus, S. epidermidis*, *S. typhi, E. coli,* S1-LF, MRSA and VRE. In case of MRSA and VRE, the compound P2 (Actinomycin X_2_) was more effective with inhibition zones in the range of 18 and 26 mm as compared to compound P3 (Actinomycin D) (17 and 25 mm) and compound P1 (Actinomycin V) (14 and 24 mm). The compounds showed significant activity against drug resistant strains which are resistant to methicillin (MRSA) (10 μg/disc) and vancomycin (30 μg/disc) (VRE) (Fig. 6, Additional file 1: Table S3).

### MIC values of purified compounds

The MIC values of the purified compounds were determined by 96 well plate method. The Actinomycin X_2_ was found to be more potent with lowest MICs as compared to actinomycins D and V. The MIC values of purified actinomycin X_2_ against test bacteria ranged between 1.95 and 15.62 μg/ml whereas those for actinomycins V and D ranged between 2.25 and 31.25 μg/ml, and 2.0 and 15.0 μg/ml, respectively. All the three actinomycins were found to be more effective against MRSA and VRE with MICs of 1.95–2.25 μg/ml and 3.5–4.0 μg/ml, respectively than against *K. pneumoniae* sub sp. *pneumoniae*, S1-LF and *B. subtilis* with MIC values of 15.0–31.5 μg/ml, 14.23–15.90 μg/ml and 8.0–15.62 μg/ml, respectively. (Additional file 1: Table S3).

## Discussion

The emergence and spread of multidrug resistant bacteria cause an array of health problems due to various interconnected factors, many of which are related to over and misuse of antimicrobial drugs and acquisition of resistance genes [34–36]. The rising levels of antibiotic resistance have complicated the treatment therapy for HAI (health care–associated infections) MRSA and VRE infections [37]. In this biological arm race, humans appear to be helpless as pathogens continue to develop resistance against each new drug introduced in the market. Hence, there is an urgent need to search new antimicrobial agents against these pathogens.

In the light of this, during our research studies to find new antibacterial compounds, a *Streptomyces* strain, designated M7, possessing potent antibacterial activity against various bacteria including MRSA and VRE was identified as *Streptomyces antibioticus*. The antibacterial compounds purified from strain M7, belonging to phenoxazone group of chromopeptides, were identified as actinomycins V, X_2_ and D on the basis of their LC-MS, NMR and FTIR spectral data [38–46]. Many *Streptomyces* spp. *viz.* *Streptomyces* MITKK-103, *Streptomyces padanus* JAU4234, *Streptomyces elizabethii, Streptomyces* sp. MS449, *Streptomyces* sp. HUST012, *Streptomyces heliomycini* are reported to produce actinomycins D, V and X_2_ simultaneously [40, 41, 47–50]. Recently, Wang et al. (2018) demonstrated the production of two new natural actinomycins, neo-actinomycins A and B formed from actinocin chromophore of actinomycin D (by the condensation of actinomycin D with α-ketoglutarate and pyruvate), in addition to actinomycin D and X_2_ from a marine-derived *Streptomyces* sp. IMB094 [51].

Actinomycins are cytotoxic compounds which exhibit potential cytotoxicity against various cancer cell lines but low toxicity against normal human cell lines [50]. They are one of the oldest anticancer drugs used in the treatment of various sarcomas. However, antimicrobial activities of actinomycins against pathogenic bacteria, especially MRSA and VRE have gained very little attention. Khieu et al. reported antibacterial activities of actinomycin D (SPE-B5.4) and a new compound (SPE-B11.8) purified from an endophytic *Streptomyces* sp. HUST012 against a set of test bacteria [*E. coli* ATCC 25922, *K. pneumoniae* sub sp. *pneumoniae* ATCC 13883, methicillin-resistant *Staphylococcus epidermidis* ATCC 35984 (MRSE) and methicillin-resistant *Staphylococcus aureus* ATCC 25923 (MRSA)]. The compound SPE-B11.8 exhibited moderate antibacterial activity with MIC values ranging between 15.63–62.5 μg/ml whereas actinomycin D showed strong activity with MIC values between 0.04–2.24 μg/ml against various test organisms [49]. Wang et al. determined antibacterial activities of actinomycins X_0β_, X_2_ and D purified from *Streptomyces heliomycini* strain WH1 and demonstrated strong activity of actinomycins X_2_ and D against *S. aureus*, methicillin-resistant *S. aureus*, *B. subtilis* and *B. cereus* with MIC values of 0.04–0.15 μM, whereas act X_0β_ displayed weak activity with MIC values of 0.3–2.5 μM [50]. Recently, Wang et al. evaluated antibacterial activity of actinomycins D, X_2_, and two new natural neoactinomycins A and B against various strains of *E. coli, K. pneumoniae,* MRSA and VRE. Actinomycins D and X_2_ were found to be very effective against MRSA and VRE with MIC values of 0.125–0.25 μg/ml, whereas neo-actinomycins A and B showed moderate to weak antibacterial activity with MIC values of 16–64 μg/ml and 128 μg/ml, respectively against MRSA and VRE. However, all the actinomycins showed weak activity against different strains of *E. coli* and *K. pneumoniae* with MIC values > 128 μg/ml [51].

In contrast, Kulkarnia et al. demonstrated antifungal activity of actinomycin D purified from an agricultural soil bacterium *Streptomyces hydrogenans* IB310 against fungal phytopathogens in addition to bacterial cultures which suggests the future application of actinomycin D in agriculture to control fungal plant diseases [52].

Our study demonstrated strong antibacterial activity of actinomycins D, X_2_ and V isolated from *Streptomyces* strain M7 against VRE, MRSA, *B. subtilis, K. pneumoniae* sub sp. *pneumoniae*, *S. epidermidis*, *S. typhi, E. coli,* S1-LF and *S. aureus.* The MIC values of actinomycins against VRE were 1.95–2.0 μg/ml, which are higher than those reported in earlier studies [49–51]. However, actinomycins were found to be more effective against *E. coli* and *K. pneumoniae* (MIC values 15.65–64 μg/ml) as compared to actinomycins D and X_2_ (> 128 μg/ml) reported by Wang et al. [51]. The findings of the present investigation also support the extended application of actinomycins to treat bacterial infections caused by drug resistant bacteria, especially methicillin-resistant *S. aureus* and vancomycin resistant *Enterococcus.*

## Conclusions

The results of the present study reveal the potential of actinomycins V, X_2_ and D, which are generally used as anticancer drugs, to treat nosocomial infections caused by various bacteria *viz.* *K. pneumoniae* sub sp. *pneumoniae*, *S. epidermidis*, *S. typhi, E. coli,* S1-LF, *S. aureus*, MRSA and VRE.

## Additional files

Additional file 1: **Table S1** Cultural characteristics of *Streptomyces antibioticus* strain M7 on different media. **Table S2** Antibacterial activity of purified compounds. **Table S3** MIC of purified compounds. (DOCX 17 kb)

Additional file 2: **Figure S1** HPLC chromatogram of purified compounds from *S. antibioticus* strain M7: (**a)** fractions (27–35), **b)** mixture of compounds P2 and P3, (**c)** compound P1**, (d)** compound P2, (**e)** compound P3. **1:** Compound P1, **2:** Compound P2, **3:** Compound P3. **Figure S2 (a)** Thin layer chromatography of *Streptomyces* M7 crude extract (**c**), mixture of purified compounds (M) and purified compounds (P). (**b)** Bioautography of purified compounds of *S. antibioticus* strain M7 against *B. subtilis.* **Figure S3** FT-IR Spectrum of purified compounds (**a)** Actinomycin V (P1), (**b)** Actinomycin X_2_ (P2), (**c)** Actinomycin D (P3). (DOCX 1971 kb)

Additional file 3: **Figure S4 (a-d):** ^1^H NMR spectra of purified Actinomycin V (P1). **Figure S5 (a-f):** ^1^H NMR spectra of purified Actinomycin X_2_ (P2). **Figure S6 (a-g):** ^1^H NMR spectra of purified Actinomycin D (P3). (DOCX 1407 kb)

## Abbreviations

DAP: Diaminopimelic acid
FT-IR: Fourier transformation infrared spectroscopy
ISP: International *Streptomyces* Project
MHA: Mueller Hinton Agar
MIC: Minimum inhibitory concentration
MRSA: Methicillin resistant *Staphylococcus aureus*
MS: Mass spectrometry
MTCC: Microbial type culture collection
NMR: Nuclear magnetic resonance
SCNA: Starch Casein Nitrate Agar
VRE: Vancomycin resistant *Enterococcus*
