# Supplementary material for: Purification and characterization of actinomycins from Streptomyces strain M7 active against methicillin resistant Staphylococcus aureus and vancomycin resistant Enterococcus
Source: BMC Microbiol. 2019 Feb 19;19:44. doi: 10.1186/s12866-019-1405-y (PMC6381723; doi:10.1186/s12866-019-1405-y)
Supplement: Supplementary file 1 — Table S1 Cultural characteristics of Streptomyces antibioticus strain M7 on different media. Table S2 Antibacterial activity of purified compounds. Table S3 MIC of purified compounds. (DOCX 17 kb) [file 12866_2019_1405_MOESM1_ESM.docx]

**Purification and characterization of actinomycins from *Streptomyces* strain M7 active against Methicillin Resistant *Staphylococcus aureus* and Vancomycin Resistant *Enterococcus***

Manish Sharma^1^ and Rajesh Kumari Manhas^1^*

^1^Department of Microbiology, Guru Nanak Dev University, Amritsar, Punjab, India

**Additional file 1**

**Table S1** Cultural characteristics of *Streptomyces antibioticus* strain M7 on different media.

| **Medium** | **Growth** | **Aerial mycelium** | **Substrate mycelium** | **Sporulation** | **Diffusible Pigmentation** |
| --- | --- | --- | --- | --- | --- |
| **SCNA** | Good | White | Yellow | Grey | Yellow |
| **ISP1** | Good | Grey | Brown | White | Light brown |
| **ISP2** | Good | White | Creamish | Grey | - |
| **ISP3** | Good | White | Yellow | Grey | - |
| **ISP4** | Good | White | Pale yellow | Light grey | - |
| **ISP5** | Good | White | White | Grey | - |
| **ISP6** | Good | White | Light brown | Dark grey | Brown |
| **ISP7** | Good | White | White | Grey | Brown |

**SCNA: Starch Casein Nitrate Agar, ISP: International *Streptomyces* Project, (-) Absent**

**Table S2** Antibacterial activity of purified compounds.

| **Test organisms** | **C**  **Crude extract** | **P1**  **Actinomycin V** | **P2**  **Actinomycin X_2_** | **P3**  **Actinomycin D** |
| --- | --- | --- | --- | --- |
|  | **Zone of inhibition (mm)*** | | | |
| ***Bacillus subtilis*** | 13±0.3 | 15±0.5 | 15±0.3 | 15±0.3 |
| ***Staphylococcus epidermis*** | 14±0.0 | 17±0.5 | 20±0.5 | 18±0.3 |
| ***Staphylococcus aureus*** | 12±0.3 | 14±0.3 | 14±0.0 | 14±0.3 |
| ***Escherichia coli*** | 13±0.5 | 14±0.3 | 13±0.3 | 15±0.3 |
| ***Klebsiella pneumoniae*** | 13±0.0 | 14±0.0 | 14±0.0 | 16±0.0 |
| ***Salmonella typhi*** | 12±0.3 | 15±0.0 | 14±0.3 | 14±0.3 |
| **MRSA** | 14±0.5 | 14±0.5 | 18±0.0 | 17±0.5 |
| **VRE** | 23±0.3 | 24±0.5 | 26±0.5 | 25±0.0 |
| ***E. coli* (S1-LF)** | 18±0.1 | 18±0.5 | 20±0.1 | 19±0.3 |

***The Values are expressed as Mean ± Standard Errors of Means (SEM) for n = 3**

**Table S3** MIC of purified compounds.

| **Treatment** | **Crude** | **P1 (Actinomycin V)** | | **P2 (Actinomycin X_2_)** | **P3(Actinomycin D)** |
| --- | --- | --- | --- | --- | --- |
| **MIC_90_ (µg/ml)** | | | | | |
| **VRE** | 2.0 | | 2.25 | 1.95 | 2.0 |
| **MRSA** | 3.95 | | 3.95 | 3.50 | 4.0 |
| ***B. subtilis*** | 15.62 | | 15.62 | 7.81 | 8.0 |
| ***K. pneumoniae*** | 31.25 | | 31.25 | 15.62 | 15.0 |
| ***E. coli* (S1-LF)** | 15.66 | | 15.90 | 14.23 | 15.53 |
