# Supplementary material for: Purification and characterization of actinomycins from Streptomyces strain M7 active against methicillin resistant Staphylococcus aureus and vancomycin resistant Enterococcus
Source: BMC Microbiol. 2019 Feb 19;19:44. doi: 10.1186/s12866-019-1405-y (PMC6381723; doi:10.1186/s12866-019-1405-y)
Supplement: Supplementary file 2 — Figure S1 HPLC chromatogram of purified compounds from S. antibioticus strain M7: (a) fractions (27–35), b) mixture of compounds P2 and P3, (c) compound P1, (d) compound P2, (e) compound P3. 1: Compound P1, 2: Compound P2, 3: Compound P3. Figure S2 (a) Thin layer chromatography of Streptomyces M7 crude extract (c), mixture of purified compounds (M) and purified compounds (P). (b) Bioautography of purified compounds of S. antibioticus strain M7 against B. subtilis. Figure S3 FT-IR Spectrum of purified compounds (a) Actinomycin V (P1), (b) Actinomycin X2 (P2), (c) Actinomycin D (P3). (DOCX 1971 kb) [file 12866_2019_1405_MOESM2_ESM.docx]

**Purification and characterization of actinomycins from *Streptomyces* strain M7 active against Methicillin Resistant *Staphylococcus aureus* and Vancomycin Resistant *Enterococcus***

Manish Sharma^1^ and Rajesh Kumari Manhas^1^*

^1^Department of Microbiology, Guru Nanak Dev University, Amritsar, Punjab, India

**Additional file 2**

**
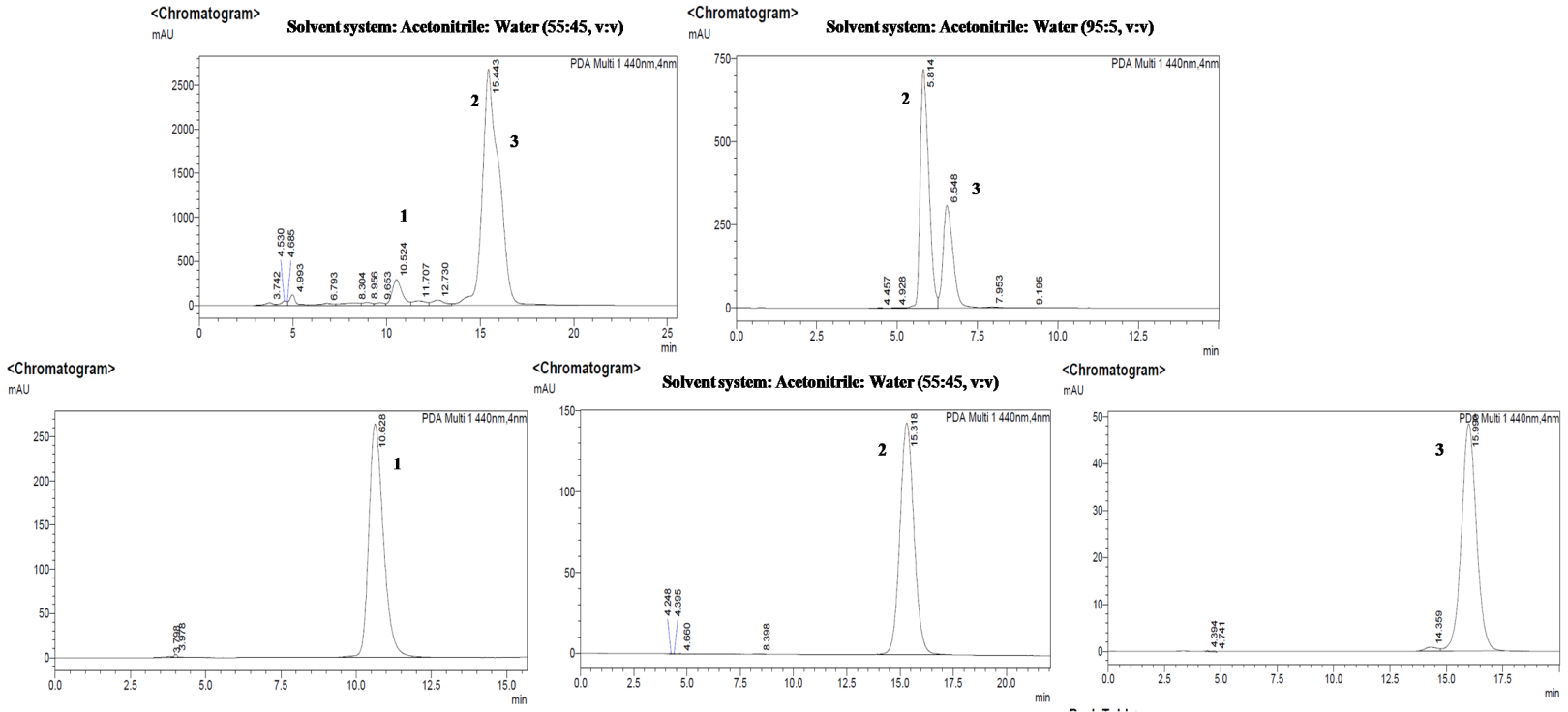
**

**Figure S1** HPLC chromatogram of purified compounds from *S. antibioticus* strain M7: (**a)** fractions (27-35), **b)** mixture of compounds P2 and P3, (**c)** compound P1**, (d)** compound P2, (**e)** compound P3. **1:** Compound P1, **2:** Compound P2, **3:** Compound P3.


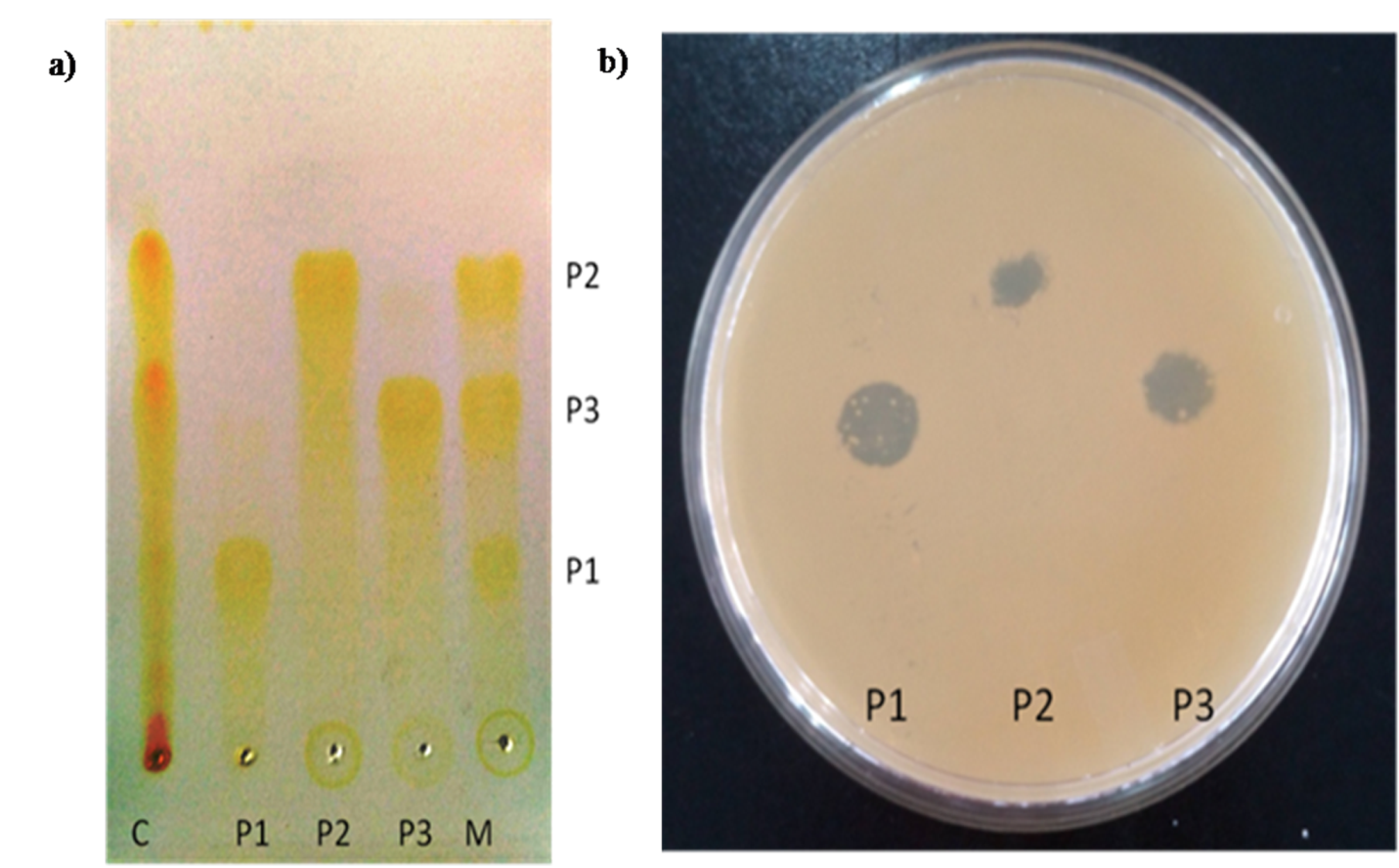


**Figure S2 (a)** Thin layer chromatography of *Streptomyces* M7 crude extract (**c**), mixture of purified compounds (M) and purified compounds (P).(**b)** Bioautography of purified compounds of *S. antibioticus* strain M7 against *B. subtilis.*


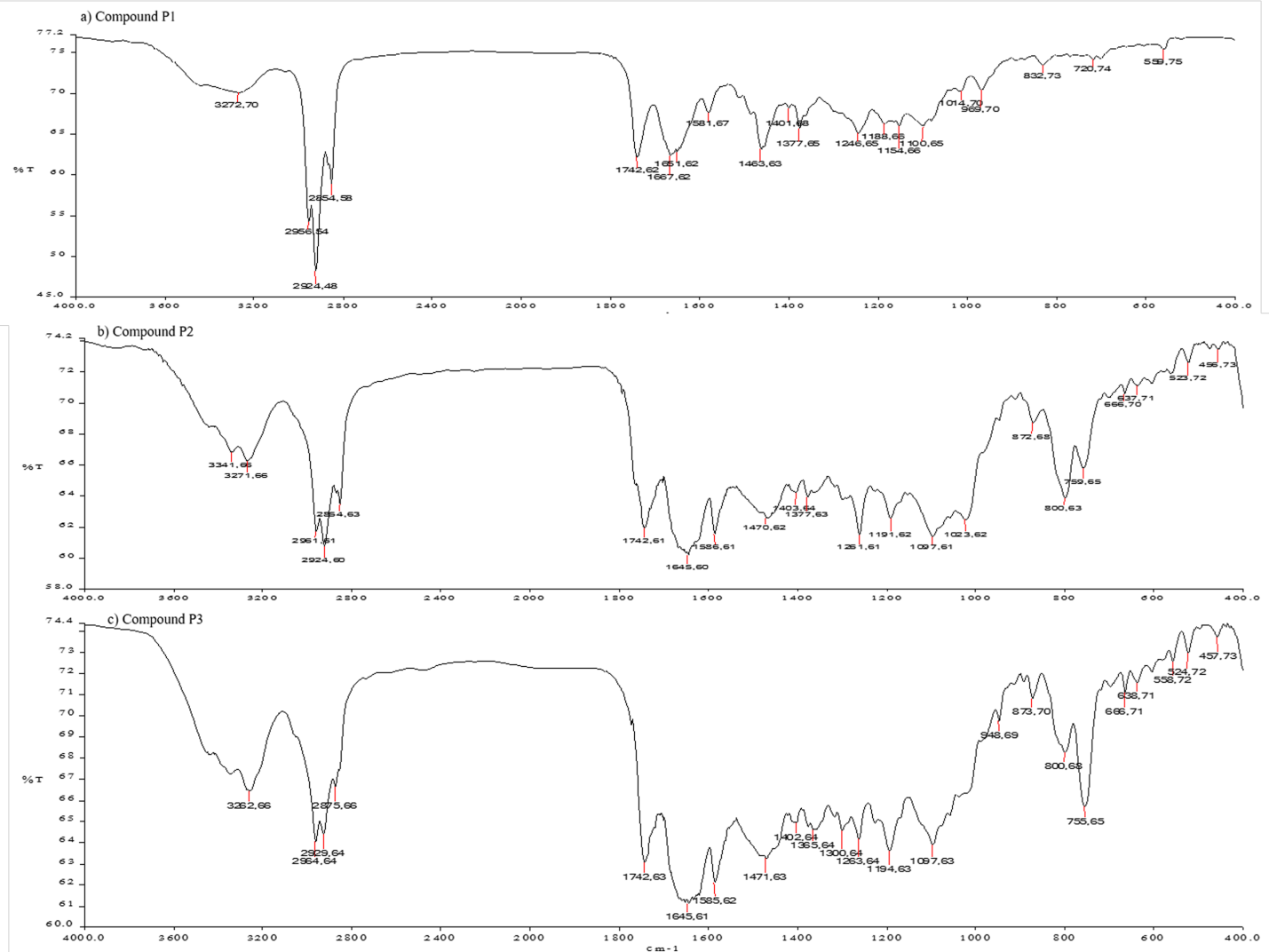


**Figure S3** FT-IR Spectrum of purified compounds (a) Actinomycin V (P1), (b) Actinomycin X_2_ (P2), (c) Actinomycin D (P3).
