# Supplementary material for: Purification and characterization of actinomycins from Streptomyces strain M7 active against methicillin resistant Staphylococcus aureus and vancomycin resistant Enterococcus
Source: BMC Microbiol. 2019 Feb 19;19:44. doi: 10.1186/s12866-019-1405-y (PMC6381723; doi:10.1186/s12866-019-1405-y)
Supplement: Supplementary file 3 — Figure S4 (a-d): 1H NMR spectra of purified Actinomycin V (P1). Figure S5 (a-f): 1H NMR spectra of purified Actinomycin X2 (P2). Figure S6 (a-g): 1H NMR spectra of purified Actinomycin D (P3). (DOCX 1407 kb) [file 12866_2019_1405_MOESM3_ESM.docx]

**Purification and characterization of actinomycins from *Streptomyces* strain M7 active against Methicillin Resistant *Staphylococcus aureus* and Vancomycin Resistant *Enterococcus***

Manish Sharma^1^ and Rajesh Kumari Manhas^1^*

^1^Department of Microbiology, Guru Nanak Dev University, Amritsar, Punjab, India

**Additional file 3**


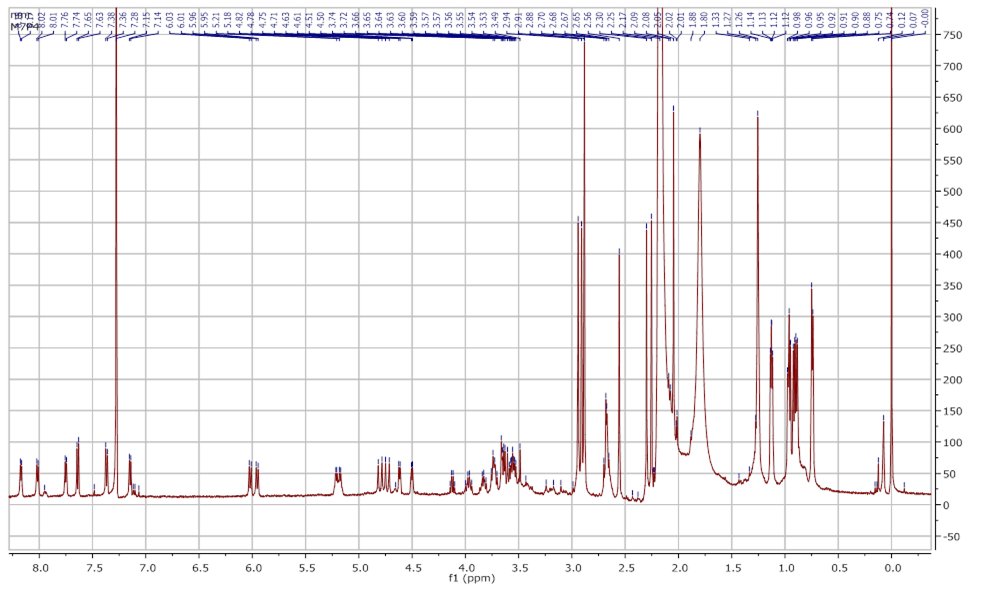


**Figure S4a** ^1^H NMR spectra of purified compounds Actinomycin V (P1).

**Figure S4b** 1H NMR SPECTRUM OF ACTINOMYCIN V (500 MHz; dCHCl3).

**Figure S4c** 1H NMR SPECTRUM OF ACTINOMYCIN V (500 MHz; dCHCl3).

**Figure S4d** 1H NMR SPECTRUM OF ACTINOMYCIN V (500 MHz; dCHCl3).


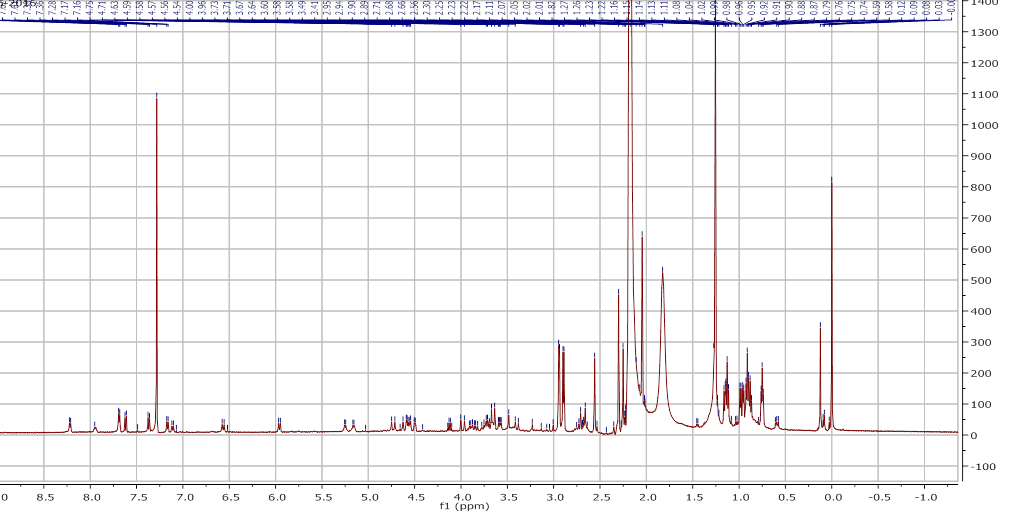


**Figure S5a** ^1^H NMR spectra of purified compounds Actinomycin X_2_ (P2) (500 MHz; dCHCl3).

**Figure S5b** 1H NMR SPECTRUM OF ACTINOMYCIN X_2_ (500 MHz; dCHCl3).

**Figure S5c** 1H NMR SPECTRUM OF ACTINOMYCIN X_2_ (500 MHz; dCHCl3).

**Figure S5d** 1H NMR SPECTRUM OF ACTINOMYCIN X_2_ (500 MHz; dCHCl3).

**Figure S5e** 1H NMR SPECTRUM OF ACTINOMYCIN X_2_ (500 MHz; dCHCl3).

**Figure S5f** 1H NMR SPECTRUM OF ACTINOMYCIN X_2_ (500 MHz; dCHCl3).


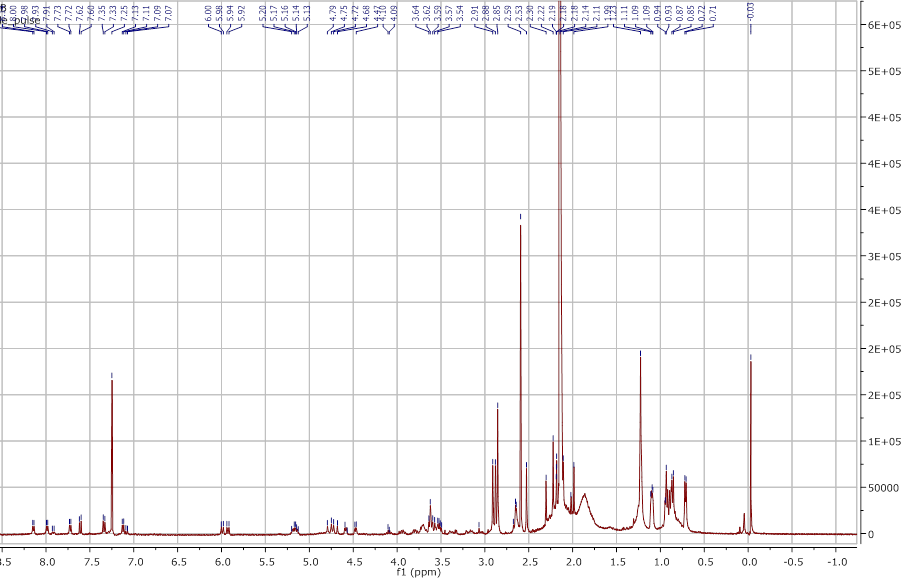


**Figure S6a** ^1^H NMR spectra of purified compounds Actinomycin D (P3) (500 MHz; dCHCl3).

**Figure S6b** 1H NMR SPECTRUM OF ACTINOMYCIN D (500 MHz; dCHCl3).

**Figure S6c** 1H NMR SPECTRUM OF ACTINOMYCIN D (500 MHz; dCHCl3).

**Figure S6d** 1H NMR SPECTRUM OF ACTINOMYCIN D (500 MHz; dCHCl3).

**Figure S6e** 1H NMR SPECTRUM OF ACTINOMYCIN D (500 MHz; dCHCl3).

 **Figure S6f** 1H NMR SPECTRUM OF ACTINOMYCIN D (500 MHz; dCHCl3).

**Figure S6g** 1H NMR SPECTRUM OF ACTINOMYCIN D (500 MHz; dCHCl3).
